# High-Intensity Virtual Reality Exergaming for Adolescents With Attention-Deficit/Hyperactivity Disorder: Protocol for a Randomized Clinical Trial

**DOI:** 10.2196/94797

**Published:** 2026-07-14

**Authors:** Martin Romero Meller, Natália Balbinot Zanini, Renato Keitel Machado, Flavia Wagner, Gabriela Bolzan, Luana Farias Oliveira Saunders, Matheus Carvalho Chagas, Luis Augusto Rohde, Pietro Merola

**Affiliations:** 1 Graduate Program in Psychiatry and Behavioral Sciences School of Medicine Universidade Federal do Rio Grande do Sul Porto Alegre, Rio Grande do Sul Brazil; 2 ADHD Outpatient Clinic Hospital de Clínicas de Porto Alegre Universidade Federal do Rio Grande do Sul Porto Alegre, Rio Grande do Sul Brazil; 3 Hospital de Clínicas de Porto Alegre Porto Alegre, Rio Grande do Sul Brazil; 4 Department of Health Medical Board and Health Division Universidade Federal do Rio Grande do Sul Porto Alegre, Rio Grande do Sul Brazil; 5 Mental Health Division Department of Health Brigada Militar do Rio Grande do Sul Porto Alegre, Rio Grande do Sul Brazil; 6 Applied Physiology and Nutrition Research Group School of Physical Education and Sport, Rheumatology Division Hospital das Clínicas da Faculdade de Medicina da Universidade de São Paulo São Paulo, São Paulo Brazil; 7 Move Sapiens Hospital de Clínicas de Porto Alegre Porto Alegre, Rio Grande do Sul Brazil; 8 Universidade Federal do ABC São Bernardo do Campo, São Paulo Brazil; 9 Department of Psychiatry School of Medicine Universidade Federal do Rio Grande do Sul Porto Alegre, Rio Grande do Sul Brazil

**Keywords:** attention-deficit/hyperactivity disorder, high-intensity interval training, virtual reality, exergaming, inhibitory control, executive function, adolescent, randomized controlled trial, exercise, inattention

## Abstract

**Background:**

Attention-deficit/hyperactivity disorder (ADHD) is a prevalent neurodevelopmental condition affecting approximately 7% to 8% of children and adolescents, characterized by persistent inattention, hyperactivity, and impulsivity. Adolescence represents a period of heightened vulnerability, during which pharmacological treatments are frequently limited by adverse effects, suboptimal adherence, and partial response. Physical exercise, particularly high-intensity interval training (HIIT), has demonstrated superior effects on inhibitory control and inattention compared with moderate-intensity continuous exercise. However, the repetitive nature and high perceived exertion of traditional HIIT protocols result in poor adherence, especially in individuals with ADHD. Virtual reality (VR)–based exergames have been proposed as a strategy to sustain vigorous physiological demands while maintaining intrinsic motivation. Despite this potential, the existing literature is predominantly limited by passive control conditions, which prevent adequate control for the effects of VR immersion and cognitive engagement, limiting causal inference regarding the specific contribution of physiological exertion.

**Objective:**

This paper presents the protocol for a randomized clinical trial designed to evaluate whether an HIIT-based VR exergame produces greater improvements in inhibitory control and inattention symptoms compared with an active, nonexercise VR control condition in adolescents with ADHD.

**Methods:**

This multisite, parallel-group, single-blind randomized clinical trial will recruit 98 adolescents aged 12 to 17 years with a confirmed diagnosis of ADHD according to the *Diagnostic and Statistical Manual of Mental Disorders, Fifth Edition*, from 2 outpatient centers in Brazil. Participants will be allocated 1:1 to an HIIT-based VR exergame intervention (Move Sapiens) or an active control condition using the same VR platform without vigorous physical exertion. The intervention comprises 20 sessions over 4 weeks delivered in a home-based format following supervised laboratory familiarization. Primary outcomes are Swanson, Nolan, and Pelham Rating Scale version IV inattention subscale scores and go/no-go commission errors. Secondary outcomes include working memory, cognitive flexibility, processing speed, impulsivity, sleep quality, and anxiety symptoms. Analyses will follow an intention-to-treat approach using linear mixed-effects models.

**Results:**

The trial is ongoing. Funding was granted in October 2024. As of April 2026, 58 participants have been enrolled across 2 sites, of whom 46 (79.3%) have completed the full intervention protocol. Data collection is expected to be completed by October 2026, with results anticipated by December 2026.

**Conclusions:**

This trial will provide controlled evidence on the efficacy of an HIIT-based VR exergame for adolescents with ADHD using an active control condition matched for technological immersion. The design will enable examination of whether vigorous physical exertion beyond VR immersion and digital engagement constitutes an essential active component for improvements in inhibitory control and inattention in this population. If effective, the intervention may offer an engaging, home-based adjunctive treatment option for adolescents with ADHD.

**Trial Registration:**

ClinicalTrials.gov NCT06632249; https://clinicaltrials.gov/study/NCT06632249

**International Registered Report Identifier (IRRID):**

DERR1-10.2196/94797

## Introduction

Attention-deficit/hyperactivity disorder (ADHD) is a prevalent neurodevelopmental condition that affects approximately 7% of children and adolescents worldwide and is associated with substantial academic, social, and functional impairment that often persists into adulthood [1,2]. Pharmacotherapy, particularly psychostimulants, remains a mainstay of treatment and is effective in reducing core symptoms [3,4]. However, functional impairments frequently persist, long-term academic and psychosocial benefits remain debated, and clinical utility is often limited by adverse effects and poor adherence during adolescence [3,5]. These limitations highlight the need for effective nonpharmacological interventions.

Physical exercise has emerged as a promising adjunctive strategy for youth with ADHD [6]. There is evidence suggesting that exercise interventions can improve specific executive function domains, particularly inhibitory control, as well as core symptoms such as inattention [7]. Nevertheless, adherence to high-intensity exercise protocols is often challenging, especially in adolescents, due to monotony and physical discomfort associated with vigorous exertion [8]. Exergaming, which integrates physical exercise with immersive digital environments, has been proposed as a means of combining physiological stimulation with enhanced engagement and motivation [9,10]. Virtual reality (VR)–based exergames in particular allow for the delivery of structured exercise protocols while maintaining high levels of user involvement.

Despite this potential, a key methodological limitation in the current literature is the difficulty of disentangling cognitive and behavioral effects attributable to physiological exertion from those related to the cognitive and motivational features of gaming and VR environments [9]. Many previous studies have relied on passive or nonequivalent control conditions, limiting causal inference regarding the specific contribution of exercise intensity [7].

To address this gap, this randomized clinical trial evaluates the effects of a high-intensity interval training (HIIT)–based VR exergame compared with an active, nonexercise VR control condition in adolescents with ADHD. The primary objective is to determine whether the HIIT-based VR intervention leads to greater improvements in inhibitory control and inattention than VR exposure without physical exertion.

## Methods

### Study Design and Setting

This study is designed as a multisite, parallel-group, single-blind randomized clinical trial with a 1:1 allocation ratio reported in accordance with the SPIRIT (Standard Protocol Items: Recommendations for Interventional Trials) 2025 guidelines (version 1.0; March 2026). The trial aims to demonstrate the superiority of the active intervention over the control condition. We will collect data at 2 research centers in Brazil: Hospital de Clínicas de Porto Alegre (Rio Grande do Sul) and Centro Escola de Especialidades Médicas in Indaiatuba (São Paulo).

### Ethical Considerations

The protocol has been approved by the ethics committee of the Hospital de Clínicas de Porto Alegre (76899123.1.0000.5327) and adheres to the Declaration of Helsinki and Brazilian General Data Protection Law. Any amendments to this protocol will be submitted to the ethics committee for approval and updated in the trial registry prior to implementation. No formal patient or public involvement was incorporated into the design of this trial. Written informed consent is obtained from legal guardians, and assent is obtained from adolescents prior to any diagnostic assessment. Participants are eligible for an incentive upon study completion: the 3 participants with the highest in-game performance scores across the intervention period will receive a license of EndeavorRx (Endeavor Digital Health).

### Participants and Eligibility

We aim to recruit 98 adolescents using social media advertising, referrals from outpatient ADHD clinics, and institutional databases. Recruitment will be balanced across the 2 study sites.

#### Eligibility Criteria

We will include participants aged 12 to 17 years with a confirmed diagnosis of ADHD according to the Diagnostic and Statistical Manual of Mental Disorders, Fifth Edition. Additionally, participants must demonstrate a score of 12 or higher on the Swanson, Nolan, and Pelham Rating Scale version IV inattention subscale. Regarding medication status, we will include participants who are either medication naive or not currently using ADHD medication (having discontinued use prior to study enrollment for at least 30 days). Importantly, the study does not request or encourage the discontinuation of prescribed medication for research purposes. However, participants who initiate or resume pharmacological treatment during the study period for clinical reasons will be withdrawn from the study to avoid confounding effects.

#### Exclusion Criteria

We will exclude adolescents who present with (1) an estimated IQ below 70; (2) a history of epilepsy, photosensitive seizures, or other neurological conditions incompatible with VR exposure; (3) medical, sensory, or motor limitations that contraindicate HIIT; or (4) severe psychiatric comorbidities, such as active psychosis or major depressive episodes with suicidal ideation. Furthermore, lack of access to a smartphone or reliable internet connection for remote monitoring or engagement in vigorous physical activity more than twice per week will result in exclusion.

### Interventions

#### Intervention Group (HIIT-Based VR Exergame)

**Figure 1:**
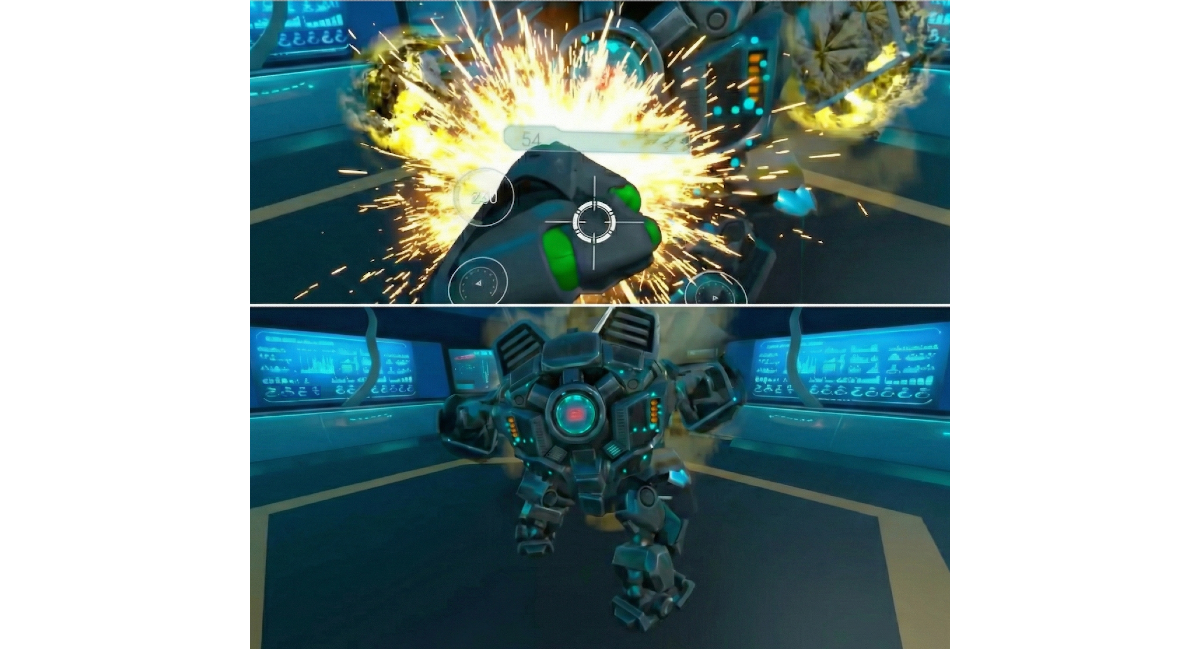
In-game user interface of the Move Sapiens virtual reality exergame (active intervention group): (A) active combat scenario with explosion and (B) environmental view.

Participants allocated to the intervention arm will engage with Move Sapiens, a VR exergame developed using Unreal Engine (version 4.27; Epic Games) and delivered through the stand-alone Meta Quest 2 and 3S headsets (Reality Labs). The game provides a fully immersive, first-person experience where players operate a virtual exoskeleton to combat an autonomous drone. The mechanics require users to simultaneously strike the drone to deplete its health, avoid incoming attacks, and continuously adjust their motor responses. This design integrates rapid motor execution, sustained attention, inhibitory control, and visuomotor coordination. The protocol consists of 20 sessions delivered over a 4-week period (5 sessions per week). Each session lasts 7 minutes and 12 seconds, adhering to a structured HIIT format composed of 12 cycles. Each cycle comprises 16 seconds of maximal physical effort followed by 20 seconds of active recovery. This specific HIIT configuration was previously validated in a pilot crossover study [11]. Results demonstrated that this protocol elicits vigorous physiological responses (mean heart rate of approximately 161 beats per minute; peak heart rate of approximately 182 beats per minute) and significant metabolic stress (lactate accumulation) while maintaining high levels of intrinsic motivation and enjoyment, supporting its utility as a potent physiological stimulus. The structure and visual interface of the Move Sapiens VR exergame are illustrated in Figure 1.

#### Control Condition (Nonexercise VR)

Participants assigned to the control group will use an adapted nonexercise version of Move Sapiens. This version preserves the same immersive VR environment, visual assets, and sound design but removes all components of physical exertion. Interaction occurs exclusively through a joystick. Session duration and frequency mirror those of the intervention group. This design enables examination of the specific contribution of vigorous physical exertion to the observed outcomes while controlling for VR immersion and digital engagement, both of which are preserved across conditions.

#### Game Design Framework

The development of Move Sapiens followed current guidelines for serious games in ADHD, particularly the IDEAL-Games framework [12], which structures development into sequential stages (idea, development, exploration, assessment of effectiveness, and long-term follow-up) and emphasizes user-centered, theory-driven design. In line with these recommendations, the game was created by a multidisciplinary team (psychiatry, exercise science, and game design), and its mechanics explicitly operationalize core cognitive targets (sustained attention, inhibitory control, and planning) through short, discrete tasks with adaptive difficulty, clear feedback, and limited session length. Reward schedules, audiovisual “juiciness,” and time constraints were planned to maximize engagement while minimizing the risk of problematic use or gaming addiction, as suggested for ADHD-oriented serious games.

### Procedures

**Figure 2:**
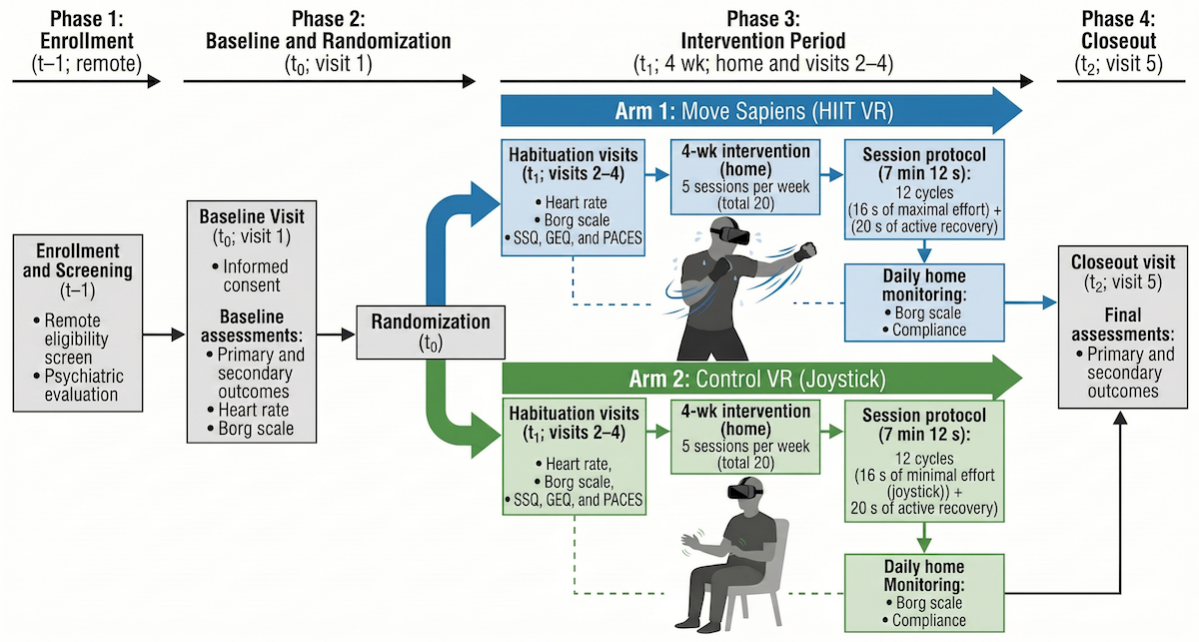
Study design flowchart and participant timeline. GEQ: Game Experience Questionnaire; HIIT: high-intensity interval training; PACES: Physical Activity Enjoyment Scale; SSQ: Simulator Sickness Questionnaire; VR: virtual reality.

The intervention follows a structured 5-week timeline comprising recruitment, laboratory adaptation, and home-based training. The workflow is divided into 4 distinct phases (Figure 2).

#### Phase 1: Recruitment and Baseline (Weeks 0 to 1)

Potential participants undergo an initial online screening. After provision of informed consent, a comprehensive psychiatric evaluation is conducted by 2 child and adolescent psychiatrists to confirm eligibility, establish the ADHD diagnosis, and screen for psychiatric comorbidities using the Schedule for Affective Disorders and Schizophrenia for School-Aged Children [13]. Eligible adolescents then proceed to the first in-person visit (visit 1), which includes the complete baseline neuropsychological assessment, physiological baseline recording (heart rate), and self-report questionnaires (Pittsburgh Sleep Quality Index and 7-item Generalized Anxiety Disorder scale). Additionally, participants experience their first exposure to the VR environment (habituation) to assess tolerability and equipment fit without engaging in the intervention protocol.

#### Phase 2: Familiarization and Safety (Week 1)

Following baseline, participants complete 3 consecutive days of supervised familiarization (visits 2 to 4) in the research center. In these sessions, participants learn to operate the Meta Quest 2 and 3S headsets and practice the specific game version corresponding to their allocation (HIIT VR or control VR). During these sessions, the research staff continuously monitors safety parameters, including heart rate (via Polar H10 sensors) and potential cybersickness symptoms. The final familiarization session includes a review of the participants’ daily routine to establish the optimal time for home practice and signing of the equipment responsibility form.

#### Phase 3: Home-Based Intervention (Weeks 2 to 5)

Participants take the VR headset home for a 4-week period. The protocol requires 1 session per day, 5 days per week (20 sessions in total). Adherence is strictly monitored via WhatsApp. Immediately after each session, participants must send (1) a screenshot of their final game score and (2) their rating on the Borg scale of perceived exertion (6 to 20). We enforce strict adherence to ensure intervention fidelity. Participants who miss a scheduled weekday session are required to make up the missed training on the weekend to maintain the total weekly dose. Three unjustified absences or failure to complete the required number of sessions despite makeup opportunities will result in exclusion from the study.

#### Phase 4: After the Intervention (Week 5)

Upon completion of the 20 sessions, participants return for the final visit (visit 5). Neuropsychological and behavioral assessments identical to those at baseline are readministered, and the VR equipment is returned.

### Outcomes

#### Overview

We will assess outcomes at baseline (visit 1) and the postintervention time point (visit 5). The specific measures are categorized as follows (Table 1).

**Table 1:** Summary of outcome measures.

Outcome domain			Instrument		Metric
**Primary**					
	Inattention symptoms	SNAP-IV^a^ inattention subscale (parent report)		Change (Δ) in total inattention score (range 0-27)	
	Inhibitory control	Go/no-go task (computerized)		Number of commission errors (responses to “no-go” stimuli); age corrected	
**Secondary**					
	Verbal working memory	Digit span task (backward condition)		Scaled score for the backward condition	
	Spatial working memory	Spatial span task (Corsi block paradigm)		Number of correct responses in the backward condition; age corrected	
	Cognitive flexibility	Trail making test (part B)		Completion time; age adjusted	
	Processing speed	2-choice reaction time task		Average reaction time for correct responses; age corrected	
	Impulsivity	MOXO-CPT^b^		Impulsivity *z*-score (standardized)	
	Sleep quality	PSQI^c^		Change (Δ) in global score (range 0-21)	
	Anxiety symptoms	GAD-7^d^		Change (Δ) in total score	
**Other prespecified domains**					
	Physiological exercise intensity	Polar H10 HR^e^ monitor (processed using the Kubios software)		Average HR, HR_max_^f^, HRR^g^ (Δ in bpm^h^ over 5 min), and HRV^i^ indexes	
	Game experience	GEQ^j^		Subscale scores and/or total experience score	
	VR discomfort	SSQ^k^		Incidence or severity of nausea, oculomotor issues, and disorientation	
	Enjoyment	PACES^l^		Total enjoyment score	

^a^SNAP-IV: Swanson, Nolan, and Pelham Rating Scale version IV.

^b^MOXO-CPT: MOXO continuous performance test.

^c^PSQI: Pittsburgh Sleep Quality Index.

^d^GAD-7: 7-item Generalized Anxiety Disorder scale.

^e^HR: heart rate.

^f^HR_max_: maximum heart rate.

^g^HRR: heart rate reserve.

^h^bpm: beats per minute.

^i^HRV: HR variability.

^j^GEQ: Game Experience Questionnaire.

^k^SSQ: Simulator Sickness Questionnaire.

^l^PACES: Physical Activity Enjoyment Scale.

#### Primary Outcomes

Primary outcomes comprise (1) inattention symptoms (assessed via the Swanson, Nolan, and Pelham Rating Scale version IV inattention subscale [parent report]), for which the primary metric is the change in the total inattention score [14]; and (2) inhibitory control (measured via a computerized go/no-go task [15]), for which the primary metric is the number of commission errors (responses to “no-go” stimuli), representing a failure in inhibitory control.

#### Secondary Outcomes

Secondary outcomes comprise (1) impulsivity (evaluated using the MOXO continuous performance test), for which the primary metric of interest is the standardized impulsivity *z*-score assessing the inhibition of impulsive responses under varying levels of environmental distractors; (2) verbal working memory (assessed using the digit span task [forward and backward conditions] to measure phonological loop capacity [16]); (3) spatial working memory (measured via the spatial span task [forward and backward conditions] assessing the ability to retain and manipulate visuospatial information [17]); (4) cognitive flexibility (evaluated using the trail making test; while both parts are administered, the outcome measure for executive shifting is the time to completion of part B [age corrected] [18]); (5) processing speed (measured using the 2-choice reaction time task analyzing both the mean reaction time and response variance [19]); (6) sleep quality (assessed via the Pittsburgh Sleep Quality Index, where a global score greater than 5 indicates significant sleep difficulties [20]); and (7) anxiety symptoms (measured using the 7-item Generalized Anxiety Disorder scale to monitor changes in anxiety levels throughout the intervention [21]).

#### Additional Measures

Additional measures comprise, first, physiological load and perceived exertion. Heart rate is monitored continuously during laboratory sessions using Polar H10 sensors. Additionally, we collect the Borg rating of perceived exertion (6 to 20) after every session (laboratory and home) to quantify training intensity [22].

The second additional measure is user experience and tolerability. VR-specific effects are assessed after exposure using the Simulator Sickness Questionnaire for adverse effects [23], the Physical Activity Enjoyment Scale for engagement [24], and the Game Experience Questionnaire for usability [25].

### Sample Size

The sample size calculation assumed a statistical power of 0.80, a significance level of an α value of .05, and a medium effect size of 0.50, consistent with literature on exercise interventions in ADHD. Accounting for an expected attrition rate of 30%, the required total sample size is 98 participants, with 49 participants allocated to each study arm. We will balance recruitment across the 2 study sites.

### Randomization and Blinding

Group allocation will be performed using a random sequence with a 1:1 allocation ratio stratified by study site. The randomization sequence will be generated by an independent researcher not otherwise involved in the study. To ensure rigorous allocation concealment and maintain the integrity of the single-blind design, the research staff is functionally divided into 2 distinct units. The blinded team includes outcome assessors responsible for baseline and postintervention assessments, as well as the data analyst, who will work exclusively with coded datasets (group A and group B) to ensure analytic neutrality. These team members have no access to the randomization list. Conversely, the unblinded team handles technical setup, safety supervision during familiarization, and daily remote monitoring as these tasks require awareness of the assigned condition.

Participant blinding to the physical nature of the task (movement vs joystick) is not feasible due to inherent differences in motor engagement. To minimize expectancy bias, participants are informed using a standardized script that the study compares “two different virtual reality training approaches for attention” without explicit mention of hypotheses regarding exercise superiority.

### Statistical Methods

All participants will be included in an intention-to-treat analysis. Continuous outcomes will be examined using linear mixed-effects models with random intercepts accounting for repeated measures and missing data. The models will include terms for treatment group, time (modeled as a continuous variable), and the treatment-by-time interaction to estimate differential change between groups. An autoregressive covariance structure will be applied to account for temporal correlation. Statistical significance will be defined as a 2-sided *P* value of less than .05. Between-group effect sizes will be estimated using the Cohen *d*. All analyses will be conducted using RStudio (Posit PBC).

### Data Management and Safety Monitoring

The REDCap (Research Electronic Data Capture; Vanderbilt University) platform will be used for data management. To ensure integrity and blinding, the blinded team enters assessment data into a restricted partition, whereas the unblinded team logs adherence and physiological data separately. All manual entries from paper forms undergo a double-check verification process by a supervisor. Data are stored on secure servers with regular backups, and the final dataset will remain locked until the analysis plan is finalized.

Regarding safety monitoring, given the low-risk nature of the exergame intervention, a formal external data monitoring committee was not convened. Instead, continuous monitoring for cybersickness and physical discomfort is conducted by the unblinded team supervisors and reviewed weekly by the steering committee. Participants reporting persistent adverse effects are withdrawn according to established safety protocols.

## Results

This trial is ongoing. Funding was granted in October 2024. Recruitment commenced in June 2024 across 2 sites: Hospital de Clínicas de Porto Alegre (Rio Grande do Sul) and Centro Escola de Especialidades Médicas (São Paulo). As of April 2026, 58 participants have been enrolled (n=35, 60.3% in Rio Grande do Sul and n=23, 39.7% in São Paulo), of whom 46 (79.3%) have completed the full intervention protocol. Data collection is expected to be completed by October 2026, with results anticipated by December 2026. No interim analyses have been conducted.

## Discussion

This randomized clinical trial evaluates the efficacy of an HIIT-based VR exergame as a novel nonpharmacological intervention for adolescents with ADHD, a population for whom effective and engaging treatment options remain limited. To our knowledge, this is the first rigorously controlled trial to investigate an HIIT-based VR intervention specifically tailored for adolescents with ADHD and delivered in a remotely monitored, home-based format. In addition to testing the clinical effectiveness of this intervention, the study incorporates a methodological design that allows for secondary exploration of whether potential benefits are primarily driven by physiological exertion, immersive VR engagement, or their interaction.

Several features strengthen the methodological rigor of this protocol. The use of an active VR control condition represents a substantial advancement over passive or nonequivalent comparators frequently used in prior studies [26], thereby improving causal inference regarding the contribution of vigorous physiological exertion beyond VR immersion and digital engagement alone to executive function outcomes. The Move Sapiens HIIT protocol has been validated via a pilot test [11], demonstrating its capacity to elicit vigorous cardiovascular and metabolic responses comparable to traditional high-intensity training while maintaining high intrinsic motivation. Focusing on adolescents aged 12 to 17 years targets a developmental stage characterized by distinctive motivational profiles and reduced adherence to pharmacological treatments. Finally, home-based delivery provides critical data on feasibility and effectiveness beyond laboratory-controlled environments. The intervention will be administered in participants’ own homes and daily routines, representing an essential step toward future clinical translation and scalability.

Several limitations should be acknowledged. The single-blind design introduces the possibility of expectancy effects; however, this risk is partially mitigated by the use of an engaging active control condition matched for novelty and immersion. Notably, expectancy bias may disproportionately affect the parent-reported inattention outcome given that parents are aware of their children’s group assignment. The inclusion of the go/no-go task as a co–primary objective performance measure allows for a complementary assessment that is less susceptible to expectancy effects as it is independent of rater expectations. Furthermore, the 2 study arms differ not only in metabolic load but also in gross motor demand and task experience as the intervention requires full-body movement whereas the control condition involves joystick-only interaction. These differences are inherent to ecologically valid comparisons of this nature and preclude definitive attribution of any observed effects to a single component of the intervention. The 4-week intervention period may be insufficient to induce durable neuroplastic changes or sustained behavioral adaptations as longer intervention durations have been associated with stronger consolidation of executive function gains in meta-analytic studies. Nevertheless, budgetary and logistical constraints limited the feasibility of implementing longer protocols, and the selected intervention length is consistent with the duration most commonly used in studies evaluating HIIT, neuromodulation, and computerized cognitive training for ADHD [27]. Excluding participants currently receiving pharmacological treatment limits generalizability but was necessary to isolate the specific effects of the exercise intervention. Although parent-reported inattention outcomes may be subject to rater bias, this limitation is offset by the inclusion of objective, performance-based measures of inhibitory control, which have demonstrated sensitivity to high-intensity exercise interventions. Finally, while home-based delivery precludes direct supervision, structured remote monitoring provides systematic indexes of adherence and training intensity, thereby ensuring intervention fidelity.

If the study demonstrates significant improvements in inhibitory control and inattention in the HIIT VR group compared with the nonexercise VR control, these findings will support the clinical efficacy of an HIIT-based VR exergame as a novel, engaging nonpharmacological intervention for adolescents with ADHD. Such results would have important implications for expanding treatment options in a population characterized by limited adherence to existing therapies. Conversely, if no differential effects are observed between groups, this would suggest that immersive VR engagement itself may represent a key therapeutic component, providing valuable insights for the design of future digital and game-based interventions. By evaluating a developmentally appropriate, home-based HIIT VR intervention within a rigorously controlled design, this trial has the potential to advance both the clinical and methodological evidence base for nonpharmacological treatments in ADHD. Results will be disseminated through peer-reviewed publications and scientific conferences regardless of the direction of the findings.

## Appendix

Multimedia Appendix 1 Peer-review report 1 from Fundação de Amparo à Pesquisa do Estado do Rio Grande do Sul (FAPERGS) and the Fundação de Amparo à Pesquisa do Estado de São Paulo (FAPESP), Brazil.

Multimedia Appendix 2 Peer-review report 2 from Fundação de Amparo à Pesquisa do Estado do Rio Grande do Sul (FAPERGS) and the Fundação de Amparo à Pesquisa do Estado de São Paulo (FAPESP), Brazil.

## Abbreviations

ADHD: attention-deficit/hyperactivity disorder
HIIT: high-intensity interval training
REDCap: Research Electronic Data Capture
SPIRIT: Standard Protocol Items: Recommendations for Interventional Trials
VR: virtual reality

## Data Availability

The datasets generated or analyzed during this study will be made available from the corresponding author on reasonable request following publication of the results.
