# Supplementary material for: High-Intensity Virtual Reality Exergaming for Adolescents With Attention-Deficit/Hyperactivity Disorder: Protocol for a Randomized Clinical Trial
Source: JMIR Res Protoc. 2026 Jul 14;15:e94797. doi: 10.2196/94797 (PMC13416302; doi:10.2196/94797)
Supplement: Multimedia Appendix 2 [file resprot_v15i1e94797_app2.pdf]

Visualizar Formulários de Avaliação

Revisor:

Avaliador 2

Proposta:

Efeitos do Exergame HIIT em VR sobre a Função Executiva em Jovens com TDAH: Um Ensaio Clínico Rando

Carregar Formulário | Imprimir | Ocultar nome do parecerista

1 - A Excelência da proposta quanto aos seguintes aspectos: qualidade e originalidade do projeto; avanço esperado em relação ao estado da arte; efetividade da metodologia proposta.: Valor entre 0 e 10 : (**9,8**) Peso: 4
[Questão Obrigatória]

2 - B Experiência prévia dos Coordenadores e equipes na área do projeto de pesquisa, considerando sua produção científica e tecnológica relevante, bem como sua contribuição na formação de recursos humanos desde 01/01/2018.: Valor entre 0 e 10 : (**10**) Peso: 3
[Questão Obrigatória]

3 - C Adequação do orçamento aos objetivos, atividade e metas propostas.: Valor entre 0 e 10 : (**9,8**) Peso: 1
[Questão Obrigatória]

4 - D Potencial de impacto dos resultados do ponto de vista técnico-científico, de inovação, difusão, socioeconômico e ambiental.: Valor entre 0 e 10 : (**10**) Peso: 1
[Questão Obrigatória]

5 - E Grau de sinergia e integração entre os trabalhos das duas equipes (RS e SP).: Valor entre 0 e 10 : (**10**) Peso: 1
[Questão Obrigatória]

6 - Detalhar os aspectos positivos e negativos da proposta (este parecer poderá ser enviado ao proponente, porém com sigilo do nome do avaliador) e justificar eventuais cortes no orçamento proposto e/ou despesas vedadas pelo edital: :
[Questão Obrigatória]

Resposta textual

O projeto é extremamente inovador e busca verificar os efeitos do exergame HIIT em VR sobre a função executiva em jovens com TDAH. A justificativa apresentada é sólida, demonstrando a relevância deste modelo imersivo somado ao HIIT em diferentes parâmetros associados ao TDAH de jovens. Sabendo a prevalência do TDAH e suas consequências quando não tratadas adequadamente, o desenvolvimento de estratégias que possam aumentar a adesão dos participantes é fundamental. Diante disto, o projeto é muito robusto metodologicamente, com um desenho experimental claro e com cuidados importantes a serem observados pela equipe. O orçamento é detalhado e mostra o que será adquirido. No entanto, para este revisor, ficou a dúvida sobre a quantidade de dispositivos VR. Pelo orçamento, serão adquiridos 10. Pensando que o projeto será realizado nos dois estados simultaneamente, e existe no planejamento, que os participantes levem para casa, como será esta logística? Me parecem poucos equipamentos. Ainda, a sinergia entre as duas equipes é muito significativa e o potencial do projeto é muito bem explorado e detalhado.

7 - Baseado na sua avaliação, seu parecer é?:
[Questão Obrigatória]

Peso: 0

- (●) *Recomendado* Valor:(0)
- ( ) *Não recomendado* Valor:(0)

Categoria	Total parcial
A Excelência da proposta quanto aos seguintes aspectos: qualidade e originalidade do projeto; avanço esperado em relação ao estado da arte; efetividade da metodologia proposta.	39,20
B Experiência prévia dos Coordenadores e equipes na área do projeto de pesquisa, considerando sua produção científica e tecnológica relevante, bem como sua contribuição na formação de recursos humanos desde 01/01/2018.	30,00
C Adequação do orçamento aos objetivos, atividade e metas propostas.	9,80

D Potencial de impacto dos resultados do ponto de vista técnico-científico, de inovação, difusão, socioeconômico e ambiental.	10,00
E Grau de sinergia e integração entre os trabalhos das duas equipes (RS e SP).	10,00
Detalhar os aspectos positivos e negativos da proposta (este parecer poderá ser enviado ao proponente, porém com sigilo do nome do avaliador) e justificar eventuais cortes no orçamento proposto e/ou despesas vedadas pelo edital:	0,00
Baseado na sua avaliação, seu parecer é?	0,00
**Total Final**	**99,00**
**Média Final**	**9,90**
